# To Break or Not to Break: The Role of TOP2B in Transcription

**DOI:** 10.3390/ijms241914806

**Published:** 2023-09-30

**Authors:** Ian G. Cowell, John W. Casement, Caroline A. Austin

**Affiliations:** 1 Biosciences Institute, The Faculty of Medical Sciences, Newcastle University, Newcastle upon Tyne NE2 4HH, UK; 2 Bioinformatics Support Unit, The Faculty of Medical Sciences, Newcastle University, Newcastle upon Tyne NE2 4HH, UK

**Keywords:** topoisomerase, DNA supercoiling, DNA topology, anticancer, DNA damage, transcription, chromatin, TOP2, TOP2B

## Abstract

Transcription and its regulation pose challenges related to DNA torsion and supercoiling of the DNA template. RNA polymerase tracking the helical groove of the DNA introduces positive helical torsion and supercoiling upstream and negative torsion and supercoiling behind its direction of travel. This can inhibit transcriptional elongation and other processes essential to transcription. In addition, chromatin remodeling associated with gene activation can generate or be hindered by excess DNA torsional stress in gene regulatory regions. These topological challenges are solved by DNA topoisomerases via a strand-passage reaction which involves transiently breaking and re-joining of one (type I topoisomerases) or both (type II topoisomerases) strands of the phosphodiester backbone. This review will focus on one of the two mammalian type II DNA topoisomerase enzymes, DNA topoisomerase II beta (TOP2B), that have been implicated in correct execution of developmental transcriptional programs and in signal-induced transcription, including transcriptional activation by nuclear hormone ligands. Surprisingly, several lines of evidence indicate that TOP2B-mediated protein-free DNA double-strand breaks are involved in signal-induced transcription. We discuss the possible significance and origins of these DSBs along with a network of protein interaction data supporting a variety of roles for TOP2B in transcriptional regulation.

## 1. Introduction

**Figure 1:**
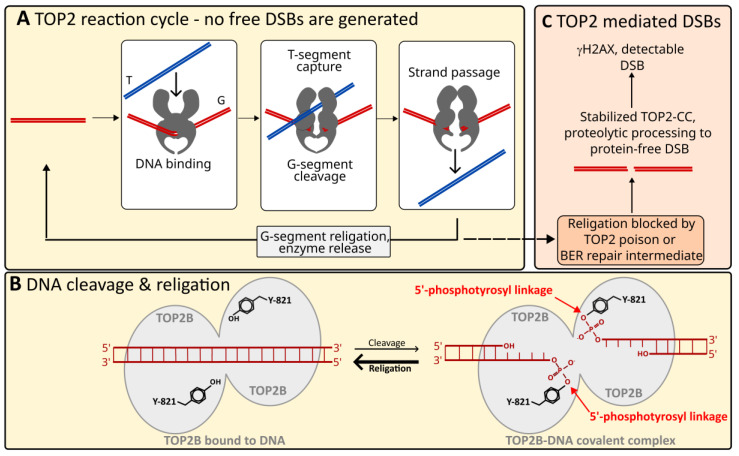
TOP2 strand passage and strand breaks. (**A**) Simplified representation of the TOP2 reaction cycle. The TOP2 dimer (grey) binds one double-stranded DNA segment (Gate or G segment—red), a second DNA duplex (Transported or T segment—blue) is captured and passed through a transient covalent enzyme-coupled DSB in the G segment before G segment religation and release. The transient enzyme-coupled strand breaks remain within the enzyme and the 5′-ends remain covalently coupled to the active site tyrosine of each protomer (Y821 in TOP2B) via a phosphotyrosine linkage as illustrated in (**B**); no free DSBs are generated in this process. (**B**) Representation of TOP2 DNA cleavage and religation products, TOP2 dimer represented as grey ovals. (**C**) After DNA cleavage, if religation is not completed, the TOP2 covalent complex (CC) is proteolytically processed, revealing a protein-free DSB. The TOP2 religation step can be blocked by the presence of TOP2 poisons such as etoposide or base-excision repair (BER) intermediates for example.

DNA topoisomerases are present in all organisms and catalyze strand passage reactions required to regulate DNA topology. This enables changes in DNA supercoiling state, and in the case of type 2 DNA topoisomerases such as vertebrate TOP2A and TOP2B, permits DNA (de)catenation and (un)knotting. These reactions are carried out via a transient enzyme-linked break in one DNA strand (Type I topoisomerases) or both strands (Type 2 topoisomerases). Typically, these DNA breaks remain buried within the enzyme, covalently attached to the catalytic tyrosine through a phosphotyrosine linkage, and are efficiently re-ligated by the enzyme after passage of a second strand or duplex through the enzyme-coupled break (Figure 1A,B). As a result, topoisomerase strand passage activity does not typically activate DNA damage signaling pathways and no DNA repair is required [1,2,3]. However, topoisomerases can generate more persistent DNA breaks if the religation step of their reaction cycle is inhibited. This occurs if the enzyme encounters specific types of DNA repair intermediate or drugs which permit the cleavage step but block religation (termed topoisomerase poisons, e.g., camptothecin (TOP1) or etoposide (TOP2)) [4]. For TOP2, the resulting stalled topoisomerase-DNA covalent complex (CC) is processed to a protein-free DSB (pf-DSB) which activates DNA damage signaling resulting in the phosphorylation of histone H2AX ser-139 (γH2AX, Figure 1C) [5].

Topoisomerases are involved in many types of DNA transaction, from replication and recombination to DNA repair and gene expression [6]. In transcription, topoisomerases regulate negative superhelical torsion behind and positive torsion ahead of an elongating polymerase as described in the twin domain model [2,7,8]. Accumulation of positive supercoils ahead of an elongating polymerase ultimately impedes elongation, and polymerase stalling could also occur due to the torque associated with accumulation of negative supercoiling upstream of the polymerase. Regulation of template supercoiling appears to be particularly important for the correct expression of long and highly expressed genes [2,8,9,10,11]. In addition to this general role in transcription, TOP2B has a specific role in the correct execution of transcriptional networks associated with neural and immune cell development [2,9,12,13,14]. Furthermore, TOP2B is implicated in efficient induction of gene expression by nuclear hormones and other stimuli [15,16,17,18,19,20,21], where gene activation is accompanied by the formation of promoter-associated DNA DSBs, seemingly induced directly by TOP2B. Notably, TOP2B is enriched in the promoter regions of most actively transcribed genes and is particularly associated with CTCF (CCCTC-Binding Factor)/cohesin genomic binding sites. Indeed, 50% of conserved CTCF genomic binding sites have been reported to be co-habited by TOP2B. Since CTCF is intimately involved with cohesin loops, the presence of TOP2B at these sites suggests a role in facilitating these loops or modulating transcription-induced supercoiling at loop boundaries [22,23]. Perhaps reflecting the diversity of these gene-expression roles, a wide range of TOP2 protein–protein interaction partners have been identified, including multiple factors involved in chromatin remodeling, transcriptional regulation, DNA repair and chromosome organization and structure [2]. In this article, we will elaborate on the specific roles of TOP2B in transcription focusing on the nature of the DSBs observed at activated promoters and on the reported protein interactions of TOP2B with established protein complexes and individual proteins associated with transcription-related processes.

## 2. Specific Roles for TOP2B in Transcriptional Programs

**Figure 2:**
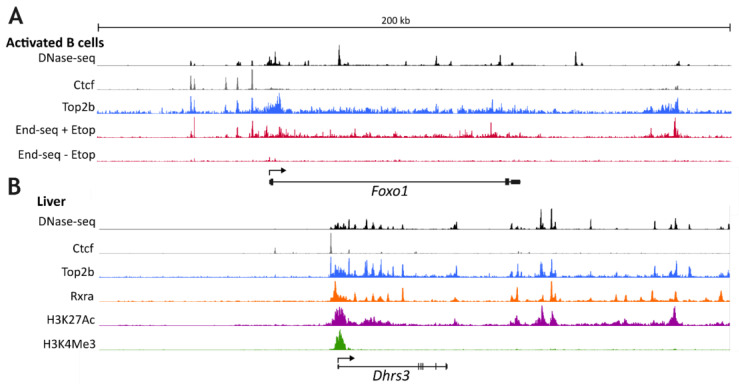
TOP2B chromatin distribution on example genes. (**A**) Distribution of DNAse-seq signal, Ctcf, Top2b and End-seq signal in the presence or absence of etoposide over the mouse *Foxo1* gene. Data are from [36] GSE172807 and [23] GSE99197. (**B**) Distribution of DNAse-seq signal, Ctcf, Rxra, H3K27Ac and H3H4Me3 in the region of the mouse *Dhrs3* gene. Data are from [36] GSE172807,GSE91731, [22] mouse liver Top2b ChIP-seq data, realigned to the mm10 genome assembly, [37] GSE197813, [36] GSE82825.

Unlike its paralogue TOP2A, TOP2B is dispensable for cell proliferation and early embryonic development [24,25]. However, while mice constitutively null for TOP2B can develop in utero, they were observed to die perinatally. This was due to failure to properly innervate the diaphragm [14]. Following this original study, others demonstrated essential roles for TOP2B in the later stages of neuronal development and in the immune system, specifically in B cell development [2,9,12,13,26,27,28,29,30]. While the role of TOP2B in these cases appears to be in establishing the correct expression of developmentally regulated genes, the mechanism/s involved are not clear. It was recently shown in a human neuroblastoma cell line model that genes downregulated in TOP2B null cells were enriched for very long genes (>200 kb) and for genes that are highly expressed in wild type cells [9]. The link with long genes and long neural genes in particular had been made previously [2,11,26,31], and suggests that TOP2B may be required for efficient transcription of long transcripts especially in post-mitotic neurons lacking TOP2A. However, since long genes are often associated with neuronal development, and some of the longest mammalian genes are neuronal specific [32], this association may reflect a particular importance of TOP2B in developmental gene expression rather than gene length. Furthermore, *PAX5*, a key B cell transcription factor gene that is under expressed in murine TOP2B hypomorphs [12], is also very long (over 185 kb). The implied requirement for TOP2B to overcome topological constraints of generating very long transcripts does not rule out involvement of TOP2B in other aspects of transcriptional regulation. For example, ChIP and ChIP combined with microarray analyses have demonstrated the presence of TOP2B at the promoters of its neural target genes in mouse tissue [33,34,35]. In fact, ChIP-seq data reveal that TOPB is generally enriched in and around promoter regions of genes [22,23], including *PAX5* [12] and other B-cell regulators, including *FOXO1* (Figure 2A). As alluded to above, a large proportion of CTCF ChIP-seq peaks correspond to peaks of TOP2B binding [22]. A pattern of TOP2B distribution observed for *PAX5*, *FOXO1* and other genes consists of a sharp TOP2B peak corresponding to a site of CTCF binding, usually upstream of the TSS, embedded in a much broader region of TOP2B enrichment extending well into the body of the gene [12] (Figure 2A).

## 3. TOP2B and Stimulus-Induced Gene Expression

We describe above how TOP2B is necessary for the correct expression of genes in certain developmental settings, but TOP2B is also required in the context of stimulus-induced gene expression. For example, TOP2B is essential for efficient nuclear hormone induced transcription, activation of *Fasn* (fatty acid synthase) upon feeding in mouse and for activation of the early-response genes including *Fos* and *Npas4* following neural activity in mouse primary neurons (see [2,9,35] and references therein). Similarly, the normally large retinoic acid-stimulated induction of genes such as *CYP26A1*, *CYP26B1*, *DHRS3* and *CRABP2* is dramatically curtailed in TOP2B null neuroblastoma cells [9]. A common feature in each of these examples is a rapid move from low to high level expression, leading to the idea that TOP2B is required in some way for the speedy changes in chromatin organization that accompany this switch in transcription state. Consistent with this, TOP2B is enriched in the 5′-end of RA inducible genes such as *Dhrs3* (Figure 2B). Intriguingly, gene activation in these cases of signal-induced transcription appears to be associated with localized TOP2 dependent DNA breaks. For example, estradiol induced induction of the *TFF1* gene, retinoic acid (RA) induced expression of *RARB,* thyroid hormone (T3) induced DIO1 and TPA induced MMP12 [15] as well as feeding induced induction of *Fasn* transcriptional activation [16] was accompanied by the appearance of a DNA break (free 3′-OH) in the promoter region of the respective genes that could be labelled with biotin-dUTP through the action of terminal deoxynucleotide transferase (TdT) [15,16]. Although this assay does not specifically detect DNA double-strand breaks (DSBs) as TdT labelling with biotin dUTP efficiently detects both nicks and DSBs, ChIP-PCR assays revealed that gene activation was accompanied by recruitment of not only TOP2B, but also the DNA DSB repair factor DNA-PK and PARP1 to the promoter region of the relevant genes [15,16]. Furthermore, in a different study [21] dexamethasone treatment resulted in promoter DSBs detected with iterative primer extension in the glucocorticoid-responsive MMTV promoter. Dexamethasone also led to the recruitment of TOP2B, and the DSB repair factors KU70 (XRCC6) and KU80 (XRCC5) to the MMTV promoter, and notably, the TOP2 catalytic inhibitor merbarone inhibited both dexamethasone-induced transcription and strand breaks [21]. In other studies, it was observed that gene activation was accompanied by localized histone H2AX phosphorylation [35,38,39], an established marker of DNA DSBs [40], supporting the notion that at least some of the signal-induced strand breaks represent DSBs. As for the MMTV example above, estradiol-induced DNA breaks in the *TFF1* promoter were inhibited by merbarone, suggesting a direct role of TOP2 in the induction of these breaks. In addition, NMDA-induced induction of *Fos* and *Npas4* and localized histone H2AX phosphorylation in cultured mouse neurons were both suppressed following shRNA-based depletion of TOP2B [35]. Furthermore, the presence of NMDA-induced DSBs in the *Fos* gene was demonstrated using the independent method of COMET-FISH [41]. The appearance of TOP2-induced breaks, including DNA DSBs that initiate local H2AX phosphorylation is on the face of it surprising, since TOP2 does not produce such protein-free breaks as part of its standard reaction mechanism. Canonically, the transient enzyme-linked strand breaks formed during the reaction cycle remain buried within the enzyme as TOP2-covalent DNA complexes (TOP2-CCs) and are rapidly resolved after strand-passage (Figure 1A,B). However, if religation is impeded, TOP2-CCs can be processed to protein free DSBs (pf-DSBs) [5] which can initiate a DNA damage response including phosphorylation of histone H2AX (Figure 1C). This leads to the question of whether these promoter-associated, signal-induced, TOP2B-dependent DSBs are the incidental consequence of occasional failure of TOP2 to quickly complete its reaction cycle or are part of a mechanism that specifically requires the formation of distinct pf-DNA DSBs for efficient transcriptional induction (see Section 6).

Other studies have linked TOP2-induced strand breaks with RNA polymerase promoter proximal pause release [38,39]. Many genes, including serum-induced immediate early genes such as *FOS*, *JUN* and *EGR1* and the heat-shock protein *HSPA1B* are regulated at this stage [42], where RNA polymerase pauses approximately 30–100 bp downstream of the transcriptional start site (TSS). Stimulation relieves the pause, allowing productive elongation to commence. In one study, phospho-H2AX and PRKDC (DNA-PK_cs_) accumulation was observed in the TSS and gene bodies of *FOS*, *JUN* and *EGR1* following release from serum starvation [43], consistent with the presence of DSBs. Notably, TOP2B also accumulated at the TSSs and the TOP2 catalytic inhibitor ICRF-193 increased pausing and led to decreased accumulation of phospho-H2AX [39]. Additional support for the association of TOP2B and DSBs in polymerase pausing, is provided by recent whole-genome DNA break analysis using the sensitive DSBCapture whole genome break mapping technique; this revealed that in unperturbed cells genomic DSBs are preferentially located around the TSSs of highly transcribed and paused genes and that pause sites are enriched in DSBs. Furthermore, treatment with the TOP2 poison etoposide (which prevents religation of the normally transient DSB, resulting in the accumulation of pf-DSBs) increased DSBs at pause sites implicating TOP2 in these DNA breaks [23,44].

## 4. TOP2B Protein Interactors

### 4.1. TOP2B/DNA-PK/PARP Connection

Several separate studies have found TOP2B to colocalize with the DNA repair proteins DNA-PK_cs_ (PRKDC), KU70 (XRCC6), KU80 (XRCC5) and PARP1 in the promoter regions of TOP2B-dependent inducible genes [2,15,16,19,20,39,45,46]. Independently, protein–protein interactions have been identified between TOP2B and PRKDC [16], XRCC6 [22,43], XRCC5 [16] and PARP1 [15,22,47,48]. Although the precise role of these repair proteins in this context remains unclear, they may relate to the induction of transcription-coupled DSBs discussed above, as it is possible to imagine that rapid repair of promoter DSBs would be required to prevent transcriptional silencing and possible genomic instability. However, it has also been suggested that the protein kinase activity of DNA-PK, activated by promoter DNA breaks and phosphorylating downstream targets, may form part of the mechanism that turns on transcription in inducible genes [16,39].

### 4.2. Broad Range of Protein Interaction Partners

**Figure 3:**
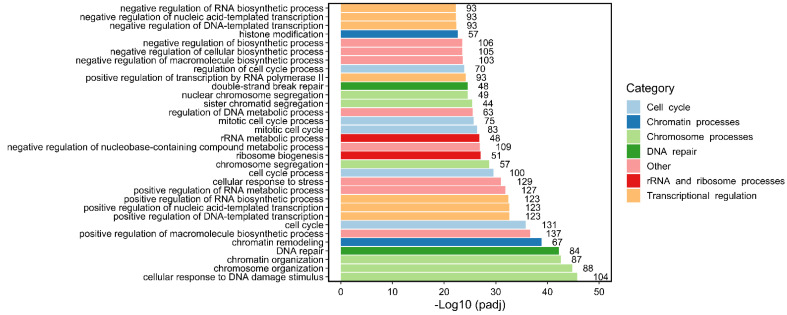
Gene set enrichment analysis (GSEA): Biological Processes most enriched amongst TOP2B interacting proteins. A non-redundant list of TOP2B interactors was compiled from previous publications and from querying BioGRID [52] and IntAct databases. This list was the input for GSEA analysis using g:GOSt functional profiling tool (https://biit.cs.ut.ee/gprofiler/gost (accessed on 10 January 2023)). The top 32 overrepresented Biological Processes (BPs) were plotted according to their significance (-Log10 multiple testing corrected *p*-value (padj)). The intersect size is shown to the right of each bar. BPs were manually placed into seven color-coded functional categories shown on the right.

**Figure 4:**
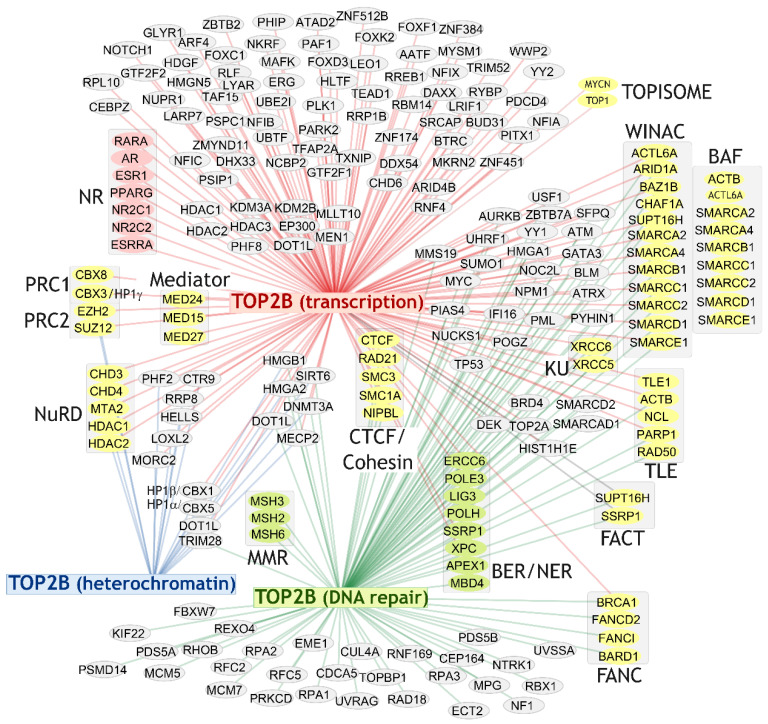
Transcription, DNA repair and heterochromatin—associated TOP2B interactors, complexes and pathways. TOP2B interactors contained in transcription, DNA repair or heterochromatin categories of Biological Processes (see Figure 3) are grouped according to their category, and further grouped (shaded boxes) according to membership of particular protein complexes (Topoisome, WINAC, BAF, TLE, FACT, NURD, PRC1/2, Mediator, KU, CTCF/Cohesin) or functional group/pathway (BER, NER, MMR, FANC, NR). Data were plotted using Cytoscape [55].

A number of TOP2B protein–protein interactions have been identified through traditional biochemical isolation of protein complexes and subsequent identification of individual components. For example, affinity purification of the TLE complex from rat neural stem cells using TAP-tagging revealed TOP2B to be part of a complex of 13 polypeptides, including non-muscle myosin II heavy chain (MYH9), β-actin (ACTB), RAD50, PARP1, nucleolin (NCL), HSP70, p54nrb (NONO), and nucleophosmin (NPMP) [45]. In a second example, affinity purification of USF1 interacting proteins from mouse liver revealed a complex containing TOP2B, PRKDC (DNA-PK), XRCC5 (KU80), XRCC6 (KU70), PARP1, PP1, KAT2B (P/CAF) [16]. Using a similar approach both TOP2A and TOP2B were found in immunoprecipitates of HA-tagged MYCN [49]. A subsequent study demonstrated that TOP2A and TOP2B appear in functional complexes with TOP1 and either MYC or MYCN respectively. The TOP2-MYC/MYCN-TOP1 complex, termed the topoisome, is proposed to resolve DNA overtwisting and supercoiling associated with high output transcription [50]. Many additional TOP2B protein interactions have been identified via high-throughput screens including proximity-labelling (e.g., BioID) [22,43,51] or IP/Affinity purification followed by mass-spectroscopy [47]. Although the significance and biological importance of such high throughput screen hits is not always certain, evidence for direct or indirect interaction with multiple components of the same complex or pathway, or statistical filtering, can lead to higher confidence. For example, a BioID screen for TOP2B-interacting proteins revealed 25 high-confidence interactors, including CTCF and several other cohesin complex components, the chromodomain protein CBX8 and topoisomerase I. Querying protein interaction databases such as BioGRID [52] and IntACT (http://www.ebi.ac.uk/intact) revealed a total of several hundred distinct TOP2B protein interactions partially derived from high-throughput screens. Biological processes relating to chromosome and chromatin processes, DNA repair, chromatin remodeling, and transcriptional regulation scored with the greatest significance in enrichment analysis of these interactions (see Figure 3), consistent with an important role in transcriptional processes. Manual inspection and comparison with the CORUM comprehensive resource of mammalian protein complexes database [53] revealed an association of TOP2B with the TLE complex as described above, but also with multiple components of the WINAC, BAF and NuRD ATP-dependent chromatin remodeling complexes and the FACT histone chaperone complex (Table 1, Figure 4). This points to a connection between TOP2B transcriptional functions and chromatin remodeling. Also of note, interactions were detected between TOP2B and three constituents of the Mediator Complex (MED15, MED24 and MED27, Figure 4) [47] which is involved in many steps of transcriptional regulation, including facilitating promoter–enhancer interactions and nuclear receptor function [54]. TOP2B interactors from the above analysis also include factors involved in transcriptional repression and heterochromatin, including members of the PRC2 polycomb complex (EZH2, SUZ12), polycomb- and HP1- class chromodomain proteins (CBX8, CBX3, CBX5). DNA repair proteins also feature in the list of TOP2B interactors. In addition to the NHEJ-associated factors PRKDC, XRCC6 and XRCC5 (DNA-PKcs, KU70 and KU80, see above), components of the base excision and nucleotide excision repair pathways were also present amongst the set of TOPB interactors (see Figure 3 and Figure 4). It is tempting to suppose that the presence of DNA repair proteins amongst the set of TOPB interactors reflects the capacity of TOP2 to generate DNA strand breaks either as an incidental aspect of its normal activity, or mechanistically where the formation of distinct DNA DSBs are required for efficient transcriptional induction.

### 4.3. TOP2B Transcription Factor Associations

The protein interaction studies described above identify a number of transcription factors that can associate with TOP2B (see Figure 4), this includes CTCF, MYCN and several nuclear hormone receptors such as RARA, AR, ESR1, ESR2 and PPARG. However, in an alternative approach to determine whether TOP2B colocalizes with specific transcription factors in chromatin we previously compared sequences contained under TOP2B ChIP-seq peaks from human MCF7 cells with motifs contained in the JASPAR database of transcription factor DNA binding sites [60,61]. Motifs corresponding to the following transcription factors were significantly enriched under TOP2B ChIP-seq peaks: CTCF, ESR1, ESR2, PPARG, TFAP2, MYF, REST, TFCP2l1, PAX5, INSM1 and AP1. The association with CTCF was later confirmed by others in mouse cells as alluded to above, where about 50% of CTCF genomic binding sites were shown to coincide with peaks of TOP2B [22,23]. Furthermore, CTCF and factors representing some of the other motifs (ESR1 and PPARG) were also present amongst the previously described TOP2B protein interactors. However, it should be noted that colocalization on chromatin does not necessarily require direct protein interaction. We subsequently carried out a similar analysis of motifs enriched under TOP2B peaks in high coverage mouse ChIP-seq data [22,62] from B cells, embryonic fibroblasts and liver and using the JASPAR enrichment tool (https://jaspar.genereg.net/enrichment/ (accessed on 29 March 2022)) [63]. Taking motifs with the highest odds ratio, present in all three sets, but not present in data from TOP2B null MEF cells yielded 158 motifs. These included motifs coinciding with CTCF, TFAP2, ESR2, REST that were also observed in the MCF7 set.

## 5. A Model for TOP2B Function in Transcription

**Figure 5:**
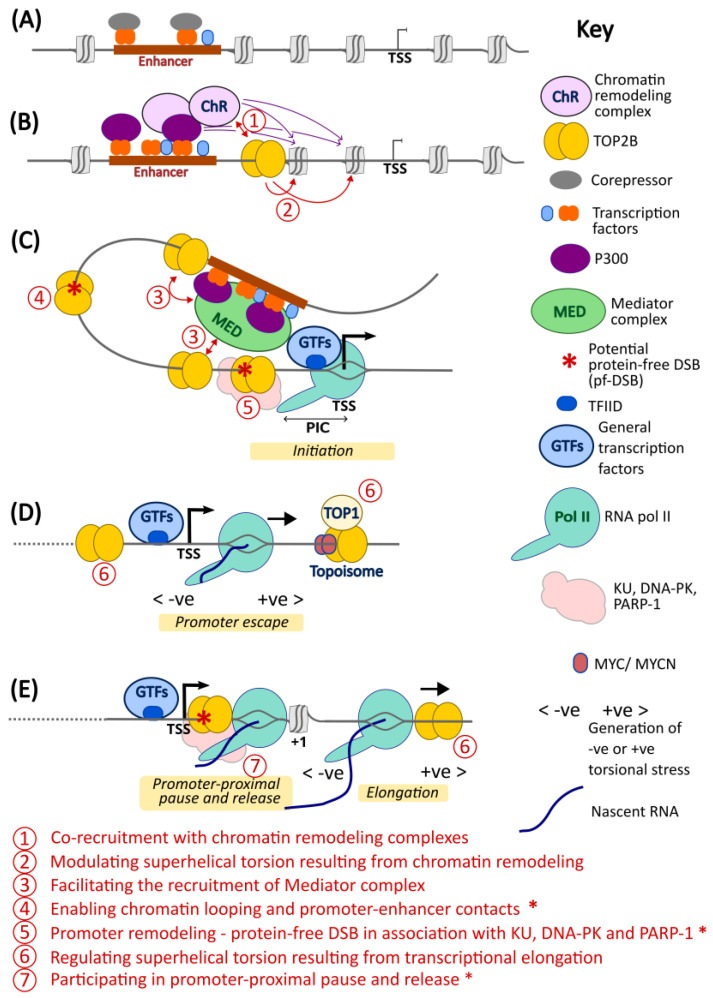
Model depicting potential modes of action of TOP2B in transcription. A simplified model for transcriptional activation by RNA pol II, highlighting points where TOP2B could potentially have a functional role (indicated by red numerals inside circles defined at the bottom of the diagram). (**A**) Gene in an “off” state, some transcription factor binding in an upstream enhancer, but these are associated with corepressors. (**B**) Early stage of transcriptional activation. Corepressors replaced by coactivators/s such as P300 along with binding of further TFs to the enhancer. The resulting histone acetylase and nucleosome remodeling activities (purple arrows) make chromatin structure more available to other factors including the basal transcription machinery but may also generate or be hindered by superhelical stress [64,65], requiring the action of topoisomerases. (**C**) Recruitment of Mediator and chromatin looping facilitates promoter–enhancer contacts, the recruitment of GTFs and PIC assembly (including RNA pol II). (**D**) Transcription initiation and promoter escape. The elongating polymerase generates +ve torsional stress ahead and -ve torsional stress behind [7,8,10]. (**E**) The initiation of transcription may be followed by transcriptional pausing a short distance downstream from the TSS [38,42]. Transcription, particularly of high output genes, generates DNA torsion and supercoiling that if not relieved by topoisomerase action could impede elongation by RNA pol II. Similarly, topoisomerases are required to manage (-) torsional stress propagating behind the early elongating polymerase into the promoter region [8,10]. The combined action of TOP1, TOP2 and MYC/MYCN in a protein complex coined the Topoisome may contribute to controlling this torsional stress [50]. Points where a pf-DSB could be envisaged to play a mechanistic role are highlighted with an asterisk.

Previous studies have demonstrated that TOP2B is required for efficient execution of certain developmental transcriptional programs and signal-induced transcription (see Section 2 and Section 3 above). While there is evidence that TOP2B-mediated DNA strand breaks may be involved (particularly in the later scenario), the precise role of TOP2 in these gene regulatory events is poorly understood. Protein–protein interaction studies, evidence for promoter recruitment of TOP2B from ChIP and ChIP-seq experiments and TOP2B-mediated promoter DSB induction (see above) together point to multiple stages in the RNA pol II transcription initiation pathway where TOP2B could play a key role. Importantly, these are not mutually exclusive possibilities. Figure 5 shows a simplified model for transcriptional activation by RNA pol II, highlighting points where TOP2B could potentially have a functional role based on available data (indicated by numerals inside circles). Firstly, the early stage of gene activation is characterized by the replacement of co-repressors by coactivators such as P300 and recruitment of chromatin-remodeling factors (Figure 5B). Protein interaction studies have demonstrated physical association between TOP2B and P300 (EP300) and between TOP2B and multiple components of ATP-dependent chromatin remodeling complexes including BAF and WINAC (Figure 4, Table 1) and with the histone chaperone complex FACT. These interactions may reflect co-recruitment of TOP2B with these complexes during transcriptional activation. In addition, the histone acetylase and nucleosome remodeling activities (Figure 5B, purple arrows) which make chromatin structure more accessible to other factors including the basal transcription machinery can also generate torsional stress resulting from the displacement or translocation of nucleosomes [64,65]. Thus, it is possible that the association of TOP2B with these chromatin remodeling factors helps put TOP2B in the correct place to manage this remodeling-linked torsion. Recruitment of Mediator and chromatin looping subsequently enables promoter–enhancer contacts, facilitating the recruitment of general transcription factors (GTFs) and pre-initiation complex (PIC) assembly (Figure 5C). Intriguingly, TOP2 has been identified as an interactor for three components of the Mediator complex (Figure 4) [47]. Although the mechanistic significance of this interaction is unknown, it is possible that it reflects stabilization or mutual stimulation of chromatin binding. Alternatively, the association with TOP2B could aid promoter–enhancer loop formation stimulated by Mediator [66], either by removing inhibitory torsional stress or via formation of a pf-DSB that facilitates the necessary rapid chromatin reconfiguration as suggested previously [35]. Transcriptional activation following a signal such as nuclear hormone ligand binding or neural activity in mouse primary neurons results in the appearance of apparently TOP2B-mediated DNA strand beaks in the promoters of target genes [15,35,41] (Figure 5 red asterisks, see Section 3). Neither the mechanistic significance nor any precise role for these DNA breaks are currently understood, but they may provide a way of quickly remodeling promoter regions to enable rapid and large magnitude changes in gene expression level. Phosphorylation of the CTD of the largest subunit of RNA pol II by CDK7 allows RNA pol promoter escape (Figure 5D) and the transition to the elongation phase of transcription. This results in the generation of negative helical torsion behind the polymerase, which is propagated into the promoter region and managed by topoisomerases including TOP2. This may be particularly significant for high-output genes [7,10]. Recently, TOP2A and TOP2B have been shown to work in concert with TOP1 and either MYC or MYCN respectively to manage transcription-associated topological stress [50]. After promoter escape, RNA Pol II often generates a short nascent RNA before pausing and awaiting further signals to enter productive transcription into the gene body (Figure 5E). This phenomenon of promoter–proximal pausing is a key transcriptional regulatory point [42]. Notably, DSB formation, at least in some cases mediated by TOP2, has been implicated in the pause release mechanism [38,39,67]. Thus, in addition to modulating elongation-associate superhelical torsion in gene bodies and promoter regions, the evidence suggests that TOP2 has a role in promoter proximal pausing and release, through a mechanism that may involve production of a pf-DSB.

High-coverage ChIP-seq analyses of TOP2B genomic distribution are limited, but from examination of available data, TOP2B is present in the 5′-regions of active genes, often in a broad area of enrichment superimposed on a sharp peak or peaks corresponding to CTCF binding (see Figure 2). In addition, End-seq, focused on DNA breaks induced by etoposide and thus measuring sites of enzymatically active TOP2, gives a broadly similar distribution [23] (Figure 2A). This is consistent with TOP2B performing an enzymatic role in this setting. This broad distribution of TOP2B chromatin occupancy and TOP2 activity does not rule out any of the possible transcriptional modes of action of TOP2B discussed above, but rather suggests that TOP2B functions at multiple points in transcriptional activation.

## 6. Conclusions and Perspective

The evidence described above supports multiple ways in which TOP2B could be required for transcriptional regulation to ensure correct gene expression. ChIP-seq analysis shows that the enzyme is enriched in gene regulatory regions [22,23] and this makes it available to solve DNA topological challenges as they arise as part of the normal processes of gene expression. This is likely to be particularly important in non-dividing cells, where TOP2A is at a low level or absent, and in situations such as nuclear hormone signaling where a rapid and large induction of transcription is required.

In addition, activation of signal-induced genes is accompanied by the appearance of TOPB-dependent DSBs. However, it is not fully understood whether DSBs themselves (as opposed to standard TOP2 strand passage activity which does not result in pf-DSBs) are required for activation under physiological conditions. Evidence supporting a central role for DSBs includes the fact that etoposide treatment can mimic aspects of signal-induced transcription and that gene activation can be accompanied by the appearance of γH2AX in promoter regions and throughout the gene body of regulated genes [35]. Although it is unclear what mechanistic role these pf-DSBs may have in transcriptional activation, it is possible to envisage models such as DSB-facilitated chromatin remodeling or enhancer–promoter looping. Furthermore, the H2AX phosphorylation associated with transcriptional activation has some curious features. Classically, induction of genomic DSBs by agents such as etoposide or ionizing radiation results in histone H2AX ser-139 phosphorylation in megabase domains flanking each DSB [40,68]. However, in the context of signal-induced transcription, the zone of ser-139 phosphorylation appears to be tightly limited to the gene body and a short distance upstream [35,39]. This possibly reflects very transient pf-DSB formation or specific features of signal-induced transcription that limit spread of H2AX-139 along the chromatin. Furthermore, DSBs arising in transcription units are generally thought to be repressive to transcription [69] rather than associated with gene activation.

It is also unclear how such TOP2B-dependent DSBs might arise since the standard strand passage activity does not produce free DNA ends (Figure 1). The appearance of DSBs that lead to H2AX phosphorylation (or can be detected by other means) points to TOP2 poisoning (i.e., a block to cleavage complex religation, requiring processing to a pf-DSB before repair (see Figure 1)). However, it is not known if this is a targeted process or a consequence of other events occurring during gene activation. For example, histone and DNA demethylation associated with a change in transcription state following nuclear hormone action generate reactive oxygen species that lead to DNA damage (oxidized bases and SSBs), requiring base excision repair [18,70]. Notably, BER-intermediates act as TOP2 poisons [4]. Thus, via the formation and processing of stabilized cleavage complexes, TOP2 can convert BER intermediates to pf-DSBs.

In most studies that report strand breaks upon stimulation of signal-induced genes the methodology used has not allowed precise location of the break. Thus, it is not known if breaks are induced at specific locations or broadly in the vicinity of the regulatory region. Nor is it clear whether the observed breaks occur at most/every active allele, or just at a minority. The answers to these questions are likely to shed light on the mode of action of TOP2B at signal-induced promoters.

Thus, it is possible that evolution has co-opted DNA breaks and DNA repair proteins to facilitate rapid changes in transcriptional state required for signal-induced transcriptional activation. TOP2B colocalizes with DNA-PK and PARP at signal induced promoters, perhaps reflecting a requirement to rapidly repair transcription-associated DSBs which would otherwise inhibit transcription [71]. But alternatively, it has been suggested that the enzymatic activities of the repair proteins may themselves facilitate steps in transcriptional activation [16,39]. More work is required to fully understand the mechanistic role of TOP2B in transcriptional regulation, both in the context of developmental programs and in signal induced transcription.

## Figures and Tables

**Table 1:** TOP2B association with chromatin remodeling complexes.

***WINAC/WSTF/ISWI Complex (CORUM:1230)—ATP Dependent Chromatin Remodeling***		
**Component**	**Synonyms**	**Reference**
ACTL6A	BAF53A, INO80K, Arp4, ACTL6	[48]
ARID1A	BAF250A, SMARCF1,	[48]
BAZ1B	WSTF (n.b. H2AX Tyr142-kinase)	[48]
CHAF1A	CAF1P150	[22,48]
SMARCA2	BRM, SNF2L2, STH1P, BAF190, SNF2	[48]
SMARCA4	BRG1, BAF190A, SNF2L4	[21,48]
SMARCB1	INI1, BAF47, SNF5L1, SWNTS1	[48]
SMARCC1	Rsc8, CRACC1, SWI3, BAF155, SRG3	[48]
SMARCC2	CRACC2, BAF170	[48]
SMARCD1	Rsc6p, BAF60A, CRACD1	[48]
SMARCE1	BAF57, CSS5	[48]
SUPT16H	CDC68, SPT16, FACTP140	[22,56]
***BAF Complex (CORUM:18)—ATP Dependent Chromatin Remodeling***		
ACTB	β-actin	
ACTL6A	BAF53A, INO80K, Arp4	
ARID1A	BAF250A, SMARCF1,	[48]
SMARCA2	BRM, SNF2L2, STH1P, BAF190, SNF2	[48]
SMARCA4	BRG1, BAF190A, SNF2L4	[21,48]
SMARCB1	INI1, BAF47, SNF5L1, SWNTS1	[48]
SMARCC1	Rsc8, CRACC1, SWI3, BAF155, SRG3	[48]
SMARCC2	CRACC2, BAF170	[48]
SMARCD1	Rsc6p, BAF60A, CRACD1	[48]
SMARCE1	BAF57, CSS5	[48]
***FACT Complex (CORUM:936)—Histone Chaperone***		
SUPT16H	CDC68, SPT16, FACTP140	[22,56]
SSRP1	FACT, FACT80, T160	[22,47]
***NuRD Complex (CORUM: 587)—Chromatin Remodeling and Histone Deacetylation***		
CHD3	MI-2A	[57]
CHD4	MI-2B	[57]
MTA2	PID	[58]
HDAC1		[58,59]
HDAC2		[58,59]

## Data Availability

Sources of previously published data are given in the figure legends including details of GEO accession numbers.
